# Pain Modulation from the Locus Coeruleus in a Model of Hydrocephalus: Searching for Oxidative Stress-Induced Noradrenergic Neuroprotection

**DOI:** 10.3390/ijms23073970

**Published:** 2022-04-02

**Authors:** Marta Louçano, Joana Oliveira, Isabel Martins, Rui Vaz, Isaura Tavares

**Affiliations:** 1 Unit of Experimental Biology, Department of Biomedicine, Faculty of Medicine, University of Porto, 4200-319 Porto, Portugal; lpm@ess.ipp.pt (M.L.); isabmart@med.up.pt (I.M.); 2 IBMC-Institute of Molecular and Cell Biology, University of Porto, 4200-135 Porto, Portugal; 3 I3S-Institute of Investigation and Innovation in Health, University of Porto, 4200-135 Porto, Portugal; 4 Chemical and Biomolecule Sciences, School of Health, Polytechnic of Porto, 4099-002 Porto, Portugal; ruivaz@med.up.pt; 5 International Doctoral School, University of Vigo, 36310 Vigo, Spain; 6 Neurosurgery Service of Centro Hospital São João, 4200-319 Porto, Portugal; jago76@gmail.com; 7 Department of Clinical Neurosciences and Mental Health, Faculty of Medicine, University of Porto, 4200-319 Porto, Portugal

**Keywords:** noradrenergic modulation, descending pain control, pain inhibition, oxidative stress

## Abstract

Pain transmission at the spinal cord is modulated by noradrenaline (NA)-mediated actions that arise from supraspinal areas. We studied the locus coeruleus (LC) to evaluate the expression of the cathecolamine-synthetizing enzyme tyrosine hydroxylase (TH) and search for local oxidative stress and possible consequences in descending pain modulation in a model of hydrocephalus, a disease characterized by enlargement of the cerebral ventricular system usually due to the obstruction of cerebrospinal fluid flow. Four weeks after kaolin injection into the cisterna magna, immunodetection of the catecholamine-synthetizing enzymes TH and dopamine-β-hydroxylase (DBH) was performed in the LC and spinal cord. Colocalization of the oxidative stress marker 8-OHdG (8-hydroxyguanosine; 8-OHdG), with TH in the LC was performed. Formalin was injected in the hindpaw both for behavioral nociceptive evaluation and the immunodetection of Fos expression in the spinal cord. Hydrocephalic rats presented with a higher expression of TH at the LC, of TH and DBH at the spinal dorsal horn along with decreased nociceptive behavioral responses in the second (inflammatory) phase of the formalin test, and formalin-evoked Fos expression at the spinal dorsal horn. The expression of 8-OHdG was increased in the LC neurons, with higher co-localization in TH-immunoreactive neurons. Collectively, the results indicate increased noradrenergic expression at the LC during hydrocephalus. The strong oxidative stress damage at the LC neurons may lead to local neuroprotective-mediated increases in NA levels. The increased expression of catecholamine-synthetizing enzymes along with the decreased nociception-induced neuronal activation of dorsal horn neurons and behavioral pain signs may indicate that hydrocephalus is associated with alterations in descending pain modulation.

## 1. Introduction

Pain is modulated by supraspinal areas which exert inhibitory and facilitatory effects on the transmission of nociceptive information in the spinal cord [1,2]. That top–down pain modulation may be altered by several conditions, such as depression and anxiety.

Hydrocephalus is a neuropathological disorder characterized by abnormalities in the flow of the cerebrospinal fluid (CSF) that results in the enlargement of the brain ventricular system [3,4,5,6]. During hydrocephalus, a compression of the periventricular tissue leads to metabolic and neurochemical changes of circumventricular brain areas [7,8]. In humans and in hydrocephalus models, motor function [3,9], as well as learning and memory [10,11] are affected. Headache pain is common during hydrocephalus, both in the initial presentation and during shunt obstruction [12]. Despite the periventricular location of some pain modulatory brain areas, it remains to be evaluated how hydrocephalus affects pain modulation from the brain, namely by the possible effects of the disease in the top–down pain control referenced above [1,2].

The main periventricular brain areas involved in descending pain modulation are the periaqueductal grey (PAG) and the locus coeruleus (LC). The PAG conveys input from cortical and subcortical regions and plays a key role in top–down pain modulation through relays in serotoninergic medullary neurons and in noradrenergic LC neurons [1]. The LC plays a pivotal role in the noradrenergic modulation of nociceptive transmission, along with the coordination of noradrenaline (NA)-mediated attention, arousal and cognition [13,14]. Disturbances of the NA-mediated actions of the LC have been associated with several neurodegenerative and psychiatric disorders [15]. The LC is an important relay nucleus in descending pain inhibition by direct noradrenergic projections to the spinal cord [16,17,18]. The activation of descending noradrenergic pathways by peripheral noxious stimulation may suppress nociceptive signals ascending from the spinal cord to the brain due to the α2-adrenoreceptor (AR)-mediated blockade of nociceptive transmission at the spinal level [16,19,20,21]. Contrary to the spinal cord, the effects of NA in the brain are complex, depending on the pain control area and the type of AR. It has been demonstrated that NA can facilitate or inhibit nociceptive transmission depending on the site of its release, the type of AR, and the intensity and duration of the pain stimulus [22,23]. Our research group has developed pioneering work in the NA-mediated enhancement of descending pain facilitation by showing that, in chronic pain models, NA released from the LC in the brain accounts for pain facilitation [2,23,24].

The main aim of present work was to study NA-mediated pain modulation from the LC and its effects on nociceptive behavioral responses and nociception-induced neuronal activation at the spinal dorsal horn in a hydrocephalus model. Despite some anecdotal references to the LC during hydrocephalic conditions [24], the LC was never evaluated in detail—specifically regarding oxidative stress—which is relevant since oxidative stress triggers NA-mediated events [25,26,27]. Moreover, local changes in NA expression affect top–down pain modulation [28], and oxidative stress is a problem in hydrocephalic patients [29]. These questions were evaluated using a validated kaolin-induced hydrocephalus model, causing ventricular and aqueductal enlargement due to oxidative stress-mediated inflammatory reaction of the circumventricular tissue [4,30,31].

## 2. Results

### 2.1. Hydrocephalic Animals Presented Increased Aqueduct and PAG Areas

**Figure 1:**
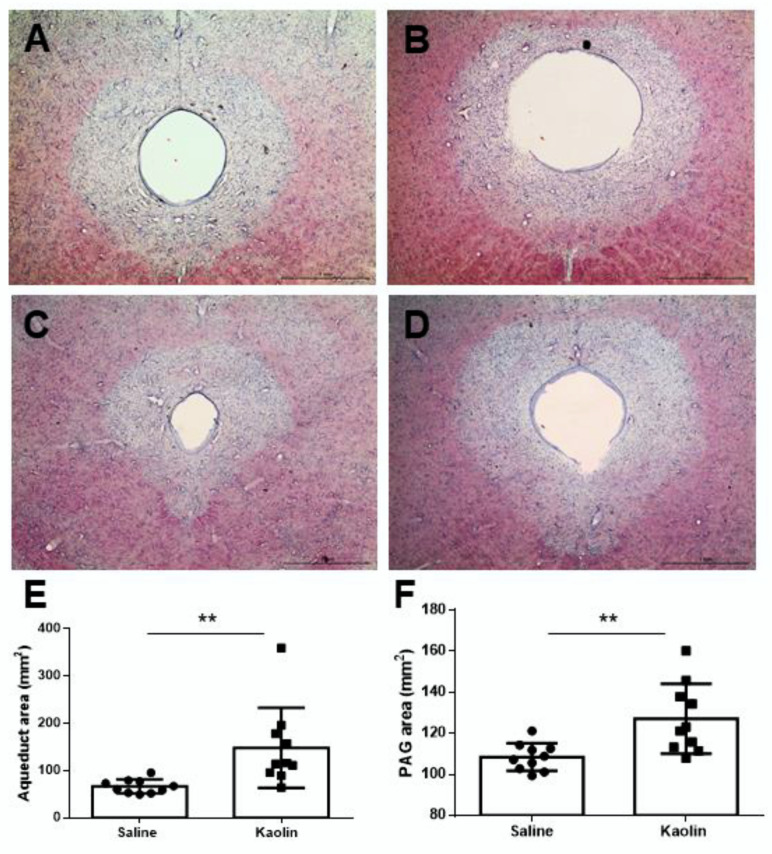
Effects of kaolin-induced hydrocephalus at the aqueduct and PAG. Representative photomicrographs of thionin-stained sections collected at about 0.70 (**A**,**B**) and 2.20 mm (**C**,**D**) rostral to the interaural line after saline (**A**,**C**) or kaolin (**B**,**D**) injections into the cisterna magna. Animals injected with kaolin presented increases in the areas occupied by the aqueduct (**E**) and PAG (**F**) areas. Saline *n* = 10; Kaolin *n* = 10. Scale bar in (**A**–**D**): 1mm. Data in (**E**,**F**) are presented as means ± SEM. ** *p* < 0.01.

Kaolin-injected animals showed a large increase in the total area of the aqueduct when compared with saline-injected animals (*p* < 0.05; Figure 1A,C,E). These animals also presented a significant increase in the area occupied by the PAG (*p* < 0.05; Figure 1B,D,F).

### 2.2. Hydrocephalic Animals Presented Increases in the Expression of Cathecolamine-Synthetizing Enzymes (Tyrosine Hydroxilase and Dopamine-β-Hydroxylase)

Locus Coeruleus: Tyrosine hydroxylase (TH), the enzyme catalyzing the rate-limiting step in catecholamine biosynthesis, is frequently used in the immunohistochemical identification of noradrenergic LC neurons [30,32] and is even considered a noradrenergic marker of the LC [31]. It was, therefore, used here to study the LC.

**Figure 2:**
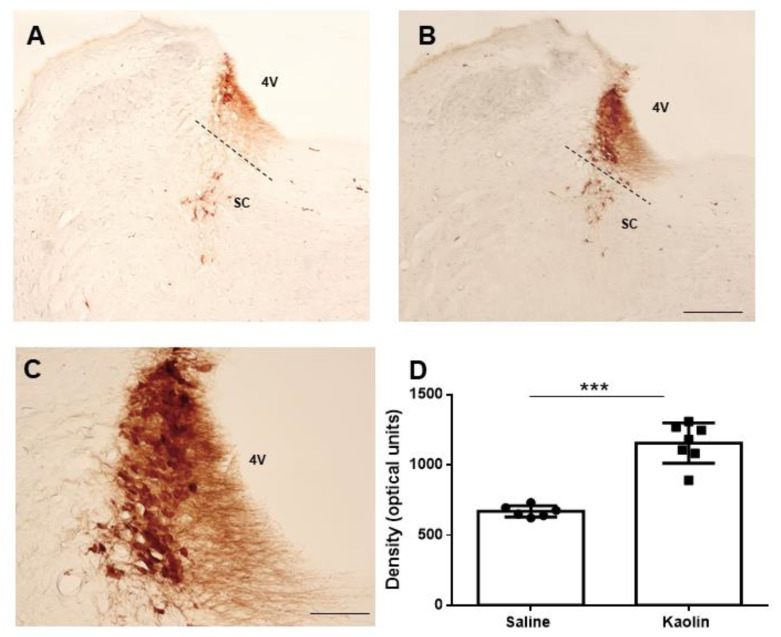
Expression of the TH at the LC. Representative photomicrographs of TH-immunolabelling in saline- and kaolin-injected animals are depicted in (**A**,**B**), respectively, from sections collected at approximately 0.80 mm caudal to the interaural line (−0.8 mm). The TH-immunoreactive neurons of the subcoerular area (SC), which is separated from the LC by the dashed line, are easier to identify but they were not counted in the present study. At the most dorsal part of the LC, individual TH-immunoreactive neurons are harder to identify due to the higher density of fibers (inset in (**C**), which is a higher magnification of (**B**)). By densitometric analysis, which analyzed both cell bodies and fibers without distinction, kaolin-injected animals presented a significant increase in TH optic density at the LC (**D**). Saline *n* = 6; Kaolin *n* = 7. Scale bar in (**B**,**C**): 1mm and 100 µm, respectively; (**A**,**B**) are at the same magnification. Data in (**D**) are presented as means ± SEM. *** *p* < 0.001.

**Figure A1:**
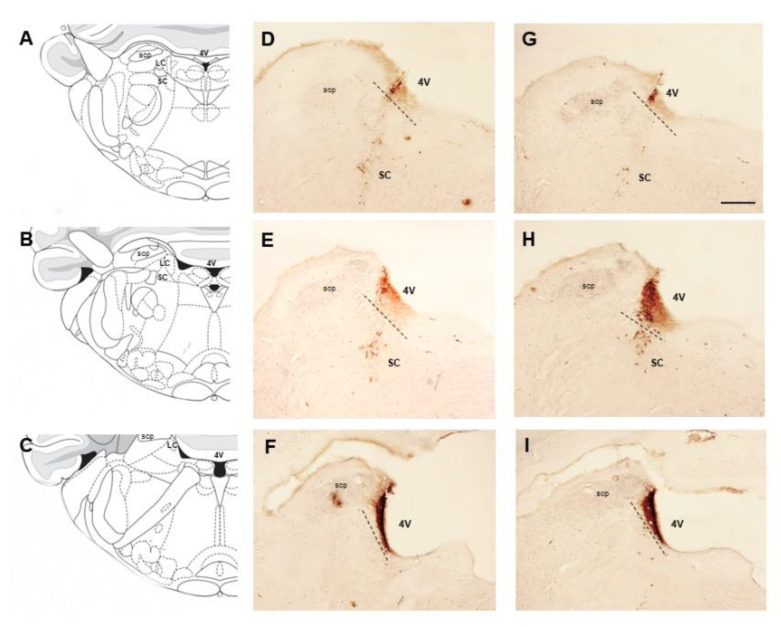
Expression of the TH at the LC. Representative photomicrographs of TH-immunolabelling in animals injected with saline (**D**–**F**) or kaolin (**G**–**I**). Each line of images was obtained approximately from the same rostrocaudal level. These levels are better depicted in the diagrams (**A**–**C**) adapted from the atlas of Paxinos and Watson, namely at 0.30 mm (**A**,**D**,**G**), 0.80 mm (**B**,**E**,**H**) and 1.30 (**C**,**F**,**I**) caudal to the interaural line. The ventricular dilation of the kaolin-injected animals frequently leads to some distortion of the 4th ventricle (4V). Additional landmarks were added, namely the superior cerebellar pedunculus (scp) and the subcoreular area (SC). In this study, the analysis was only performed at the LC area, located above the dashed lines. The differences in TH expression between the 2 experimental groups are better depicted in intermediate (−0.80 mm) and caudal (−1.30 mm), although we did not quantify in detail the density of TH-immunostaining in each rostrocaudal level. The images of the intermediate levels (−0.80 mm) are the same as Figure 2 to allow to infer the density of LC neurons at the dorsal part of the LC. Scale bar in A corresponds to 1 mm and all the photomicrographs are at the same magnification.

TH-immunoreaction at the LC was recognized by dense brown immunostaining (Figure 2A,B). The intense staining precluded the identification of neurons immunoreactive to TH (TH-IR) which was mostly due to the density of the neuropil, as described previously [33,34]. Kaolin-injected animals presented an increase in the density of TH-immunoreaction, including cell bodies and neuropil, in the LC in comparison with the control animals (*p* < 0.001). The images in Figure 2A,B depict approximately the same rostrocaudal level (around 0.8 mm caudal to the interaural line). To allow a better identification of the effects of kaolin injection in the LC, representative sections taken from three rostrocaudal levels of 6 distinct animals were included in Figure A1.

Spinal cord: The spinal cord was analyzed both for the expression of TH and of dopamine-β-hydroxylase (DBH), two of the catecholamine biosynthetic enzymes which are frequently used in the immunohistochemical studies of the noradrenergic spinal dorsal horn system.

**Figure 3:**
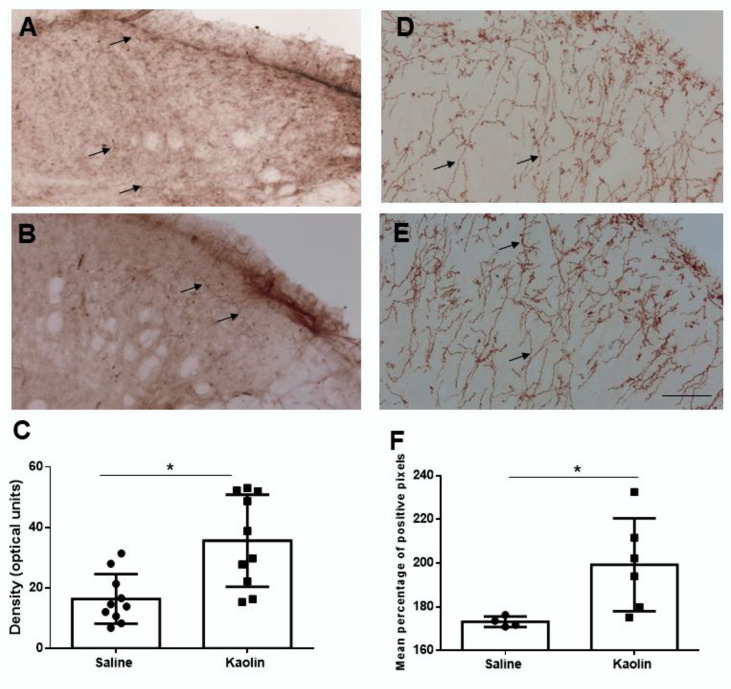
Immunohistochemical analysis of TH (**A**–**C**) and DBH (**D**–**F**) expression at the spinal dorsal horn in animals injected with saline (**A**,**D**) or kaolin (**B**,**E**). Representative photomicrographs of TH- and DBH-immunolabelling at the spinal dorsal horn are shown and the details of the immunoreactivity are pointed by the arrows, namely fibers with some varicosities. Kaolin-injected animals presented increases in TH (**C**) and DBH (**F**) expression in the spinal dorsal horn. For TH: Saline *n* = 10; Kaolin *n* = 10. For DBH: Saline: 4; Kaolin = 6. Scale bar in A: 100 µm; (**A**,**B**,**D**,**E**) are at the same magnification. Data in (**C**,**F**) are presented as means ± SEM. * *p* < 0.05.

Fibers immunoreacted (IR) for TH or DBH were distributed at the spinal cord, but the expression of DBH was more intense and widespread at the dorsal horn (Figure 3A,B). Fibers were easily recognized throughout by their conspicuous profiles with clearly identified varicosities (arrows in Figure 3A,B). Increases in the expression of TH (*p* < 0.05; Figure 3C) and of DBH (*p* < 0.05; Figure 3D) were detected in the spinal dorsal horn of kaolin-injected animals in comparison with the controls.

Collectively, the results showed higher levels of catecholaminergic enzymes both at the LC and spinal dorsal horn in kaolin-injected animals.

### 2.3. Hydrocephalic Rats Presented Increases in 8-hydroxyguanosine (8-OHdG) and Higher Co-Localization with TH at the LC

**Figure 4:**
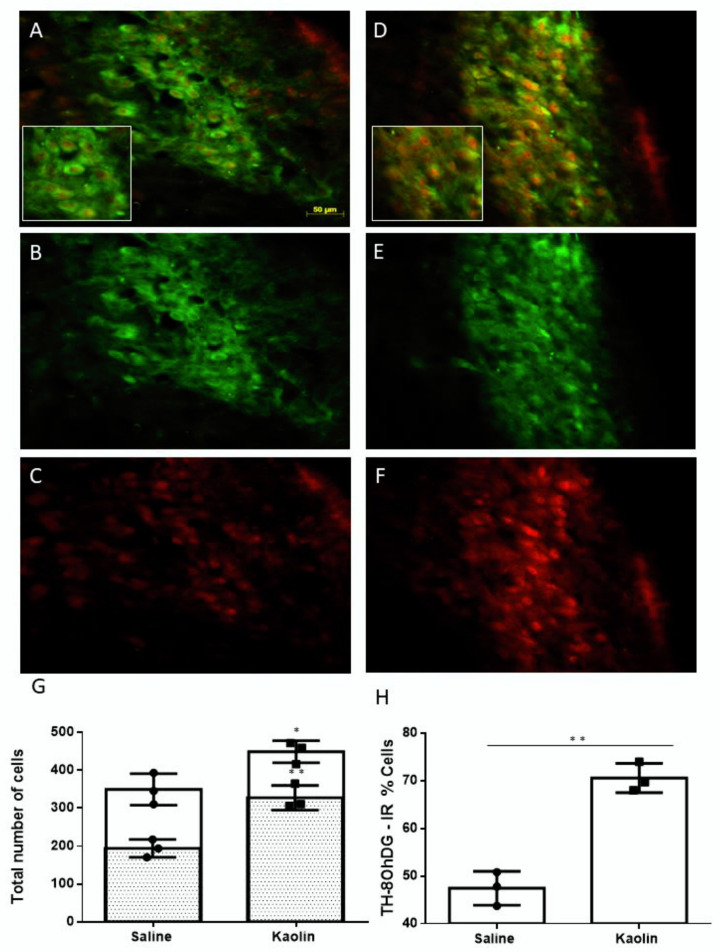
Co-localization of TH and 8-OHdG in the LC. Representative immunofluorescence photomicrographs from a saline-injected animal (**A**–**C**) and kaolin-injected animals (**D**–**F**) of the same section double-labelled for 8-OHdG and TH (**A**) or observed in a single channel for TH (**B**,**E**) or 8-OHdG (**C**,**F**). The inset in (**A**,**D**) shows the detail of neurons double-immunolabeled for TH (green) and 8-OHdG (red). Graph G shows the total numbers of 8-OHdG-IR neurons (white bars) or double-labelled for 8-OHdG and TH (grey bars). Graph H shows the percentage of TH-IR neurons that were also immunoreactive for 8-OHdG. Kaolin-injected animals presented an increase in the expression of 8-OHdG and co-localization of 8-OHdG in TH-IR neurons in the LC. Saline: *n* = 3; Kaolin: *n* = 3. Data in (**G**,**H**) are presented as means ±SEM.* *p* < 0.05; ** *p* < 0.01.

**Figure A2:**
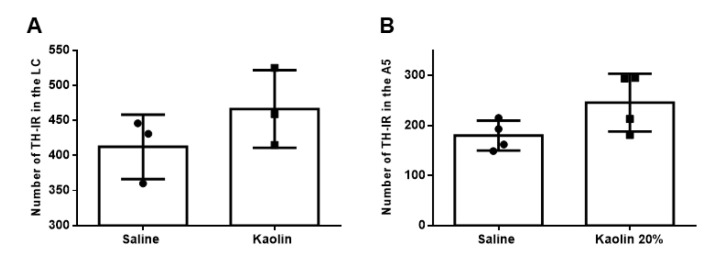
Mean numbers of TH-IR neurons in the LC (**A**) and pontine A_5_ noradrenergic cell group (**B**) of saline- and kaolin-injected animals. Counting of LC neurons was performed in immunofluorescence sections like those of Figure 4 since accurate identification cannot be performed in DAB-stained material. No statistically significant differences were detected between the 2 groups. Saline *n* = 3; Kaolin *n* = 3 in (**A**). Saline *n* = 4; Kaolin *n* = 4 in (**B**).

Neurons of double-IR for 8-OHdG-TH IR were easily recognized in the LC due to their distinct immunohistochemical profile (TH in green, 8-OHdG in red; Figure 4A–F). Kaolin-injected animals presented an increase in the absolute numbers of 8-OHdG (*p* ˂ 0.05; Figure 4G) and of neurons double-labelled for 8-OhDG and TH (*p* ˂ 0.01; Figure 4G). These animals also presented an increase in the percentage of neurons doubled for 8-OHdG and TH in the LC (*p* < 0.01; Figure 4H). The total number of TH-IR neurons at the LC were not different between groups (Saline: 412.33 ± 45.94; Kaolin: 466.33 ± 55.37; Figure A2, panel A). Collectively, the results indicated that hydrocephalic animals present a higher expression of the oxidative stress marker 8-OHdG in LC neurons and do not indicate oxidative stress-mediated neuronal loss.

### 2.4. Hydrocephalic Rats Presented Decreases in Formalin-Evoked Fos Expression in the Spinal Dorsal Horn along with Decreased Nociceptive Behavioral Responses

**Figure 5:**
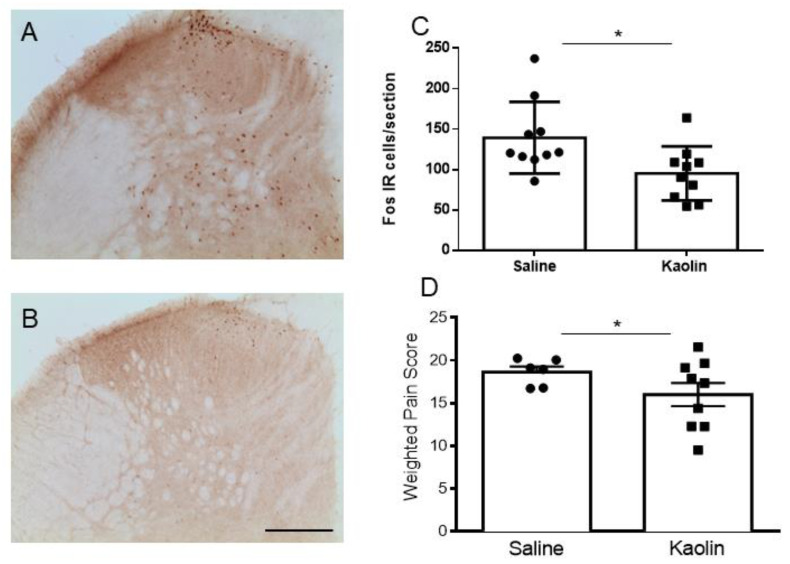
Results of the formalin-induced Fos expression (**A**–**C**) and nociceptive behaviors (**D**). Representative photomicrographs of Fos-IR neurons in the spinal cord after saline (**A**) or kaolin (**B**) injection. Hydrocephalic animals presented a significant reduction in c-Fos expression in the spinal dorsal horn (**C**). Saline *n* = 10; Kaolin *n* = 10. Scale bar: 200 µm; (**A**,**B**) are at the same magnification. Graph (**D**) shows the nociceptive behavioral responses in the second phase of the formalin test. Kaolin-injected animals showed a decrease in pain behaviors during the second phase of the formalin test. Saline *n* = 6; Kaolin *n* = 9. Data in (**C**,**D**) are presented as means ± SEM. * *p* < 0.05.

**Figure A3:**
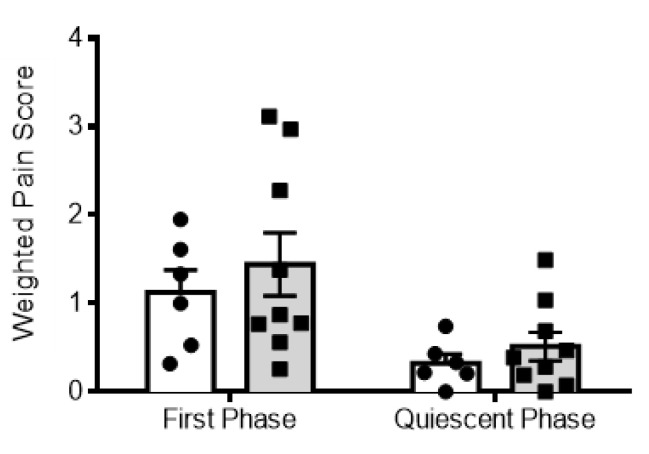
Nociceptive behavioral responses in the first and quiescent phases of the formalin test. Saline-injected (white bars) and kaolin-injected animals (grey bars) show similar pain behavioral scores, contrary to the second (inflammatory) phase of the test (*cf.* Figure 5D). Saline *n* = 6; Kaolin *n* = 9. Data is as means ± SEM.

The evaluation of formalin-induced Fos expression in the spinal dorsal horn (Figure 5A–C) showed a decrease in the numbers of Fos-IR cells in kaolin-injected animals (*p* < 0.05; Figure 5C). The subcutaneous injection of formalin produced a typical biphasic response consisting of increases in licking and biting of the injected paw and flinching of the paw and hindquarters. The first phase was short (about 10 min) and recruited mainly peripheral mechanisms, and the second phase lasted longer and sustained and recruited supraspinal mechanisms [35]. Between the two phases, a quiescent period occurred. Kaolin-injected animals presented a decrease in painful behaviors during the second phase of formalin test (*p* < 0.05; Figure 5D). No differences were detected in the first phase or quiescent phases of the formalin test (Figure A3).

Overall, the results indicated that hydrocephalic animals present reduced nociception-induced neuronal activation at the spinal dorsal horn and reduced nociceptive behaviors, in comparison with controls.

## 3. Discussion

The present study has demonstrated, for the first time, that in the kaolin-induced hydrocephalus model there was an increase in the levels of cathecolaminergic-synthetizing enzymes, both at the LC and spinal dorsal horn, and a decrease in the nociceptive activation of spinal cord neurons along with lower nociceptive behavioral responses. A marked increase in oxidative stress at noradrenergic LC neurons was also detected. The increase in the PAG area was not reported in previous studies probably because the PAG is seldomly measured and is only evaluated using imaging techniques, which do not allow precise delimitation of this small area. Whether reactive gliosis and inflammation previously reported in other brain areas of hydrocephalic animals [6] may account for the enlargement of the PAG deserves evaluation.

The increase in the levels of the enzyme TH, frequently used as a validated method to identify noradrenergic LC neurons [30,31], has never been reported in the kaolin-induced hydrocephalus model. To perform a correct counting of the TH-IR neurons in the LC, a specific processing of brainstem tissue should be performed to allow precise morphometric analysis, such as the stereological technique [30]. The high density of neurons and fibers in the LC [33,34] is known to preclude precise counting of TH-IR neurons, and densitometric analysis is frequently used in this situation. Counting the numbers of TH-immunoreactive neurons, along with an analysis of TH expression in axonal profiles, is important to infer information regarding the changes in production versus transport in this animal model. This method could also allow us to better evaluate if there are any rostrocaudal differences in the LC concerning the effects of kaolin injection. In hydrocephalus models, decreases in surrogate markers of NA levels were reported at the cortex and hippocampus [35,36], whereas the opposite was shown at the striatum [35,37]. Since the LC is the main source of noradrenergic fibers in the brain, it will be important to ascertain if there are changes in noradrenaline transport in the brain during hydrocephalus [36]. It needs to be ascertained if there are alterations in NA production and/or distribution to the brain and spinal cord. The severe structural changes in the brain of hydrocephalic animals [38] may preclude measurements of NA levels or its metabolite MHPG (3-methoxy-4-hydroxyphenylglycol), even in large brain areas. As for the spinal cord, its laminar organization frequently impairs the evaluation of the existence of alterations in neurotransmitter levels when there are expression increases in one laminae and the opposite effect in adjacent laminae [39].

An increase in both TH and DBH-IR fibers was detected in the spinal dorsal horn of hydrocephalic rats. Although the expression of those cathecolamine-synthetizing enzymes is frequently used in studies to indicate NA expression [40,41,42,43,44], it is important to perform direct NA measurements at the spinal cord. Noradrenergic innervation of the spinal dorsal horn in the rat strain used in the present study mainly originated from the LC, since around 85% of all noradrenergic neurons projecting to the spinal dorsal horn are located at the LC. It is possible that the increases in the expression of cathecolamine-synthetizing enzymes in the spinal dorsal horn are related, but this possibility needs to be ascertained by evaluating if the distribution of NA in the spinal cord is impaired in this animal model. Besides the major contribution of the LC to the noradrenergic innervation of the spinal dorsal horn, the remaining noradrenergic sources of fibers in the spinal dorsal horn originate from the pontomesencephalic A5 and A7 noradrenergic cell groups [18]. We initially counted the numbers of TH-IR neurons at the A5 noradrenergic cell group, and no differences were detected between controls and hydrocephalic animals (Figure A2, panel 2). These results suggest that the increases in TH levels at the LC in the hydrocephalus are probably mainly due to the circumventricular location of the nucleus. A detailed analysis of TH expression in all the noradrenergic cell groups in the brain, namely concerning their distance to the ventricular system, is necessary to evaluate that hypothesis.

It remains to be ascertained if the increase in the expression of the cathecolaminergic enzymes TH and DBH account for the reduced activation of dorsal horn neurons along with decreased behavioral nociresponses in the formalin test in hydrocephalic animals. We elected the formalin-induced activation of the c-Fos protooncogene at the spinal cord, since it is considered a more functional method that allowed us to use the same animal to study behavior and Fos expression, and there is usually a correlation between nociceptive activation and behavioral responses [45,46]. It is important to perform intrathecal deliveries of α2-adrenoreceptors agonists to evaluate if the function of this receptor is preserved at the spinal cord. Noradrenaline was shown to be exclusively inhibitory by binding to α2-adrenoreceptors and inhibiting the release of excitatory neurotransmitters [22]. Intrathecal administrations imply placement of a catheter for drug administration in the spinal cord, and this can cause injury or tissue compression which precludes the immunohistochemical recognition of Fos neurons.

As for the formalin test, the decrease in responses in the second phase of kaolin hydrocephalus rats supports the proposal that pain modulation from the brain is affected in hydrocephalic rats, since changes in the second phase of the formalin test are mainly associated with supraspinal modulation [23,47]. Indeed, the second phase of the test was previously shown to depend on top–down descending modulation, and is related to spinal neuronal responsiveness [46,48]. Supporting decreases in nociceptive transmission in hydrocephalic animals, nociception-induced neuronal activation at the spinal dorsal horn was decreased, as shown by the lower the numbers of Fos-IR neurons. This is important since this animal model presents motor impairments, and the evaluation of nociceptive behaviors is challenging. However, no changes in the first and quiescent phase of the formalin test were detected (Figure A1) which would be expected if the behavioral results in the second phase were due to the motor impairment.

The increases in TH at the LC of hydrocephalus rats may be explained by the high levels of oxidative stress detected in local neurons. Due to the obstruction of the connection between the ventricular system and the intracranial and subarachnoidal space and obstruction of CSF circulation [4,30,31,40], some brain areas may be hypoxic. In hypoxic situations, there is an increase in the number of reactive oxygen species (ROS) leading to an oxidative imbalance and consequently to a state of oxidative stress. The present study demonstrates, for the first time, that TH-IR neurons of the LC present increases in a validated oxidative stress marker, 8-OHdG. The role of NA in providing neuroprotection against oxidative stress has emerged [49]. Departing from initial in vitro studies showing a neuroprotective role of NA from oxidative stress [50], it was recently shown that oxidative stress-mediated changes in LC neurons during the very early phases of Alzheimer’s disease may lead to changes in local noradrenaline levels [51,52]. It is therefore important to evaluate if the increases in NA levels at the LC in hydrocephalic rats represent a neuroprotective response to oxidative stress in that periventricular region. Herein, changes in nociceptive responses in hydrocephalus rats are likely to be a subproduct of the oxidative stress-mediated increases in noradrenaline levels at the LC. The evaluation of oxidative stress levels in other brain regions should be performed. We will evaluate if intraventricular infusions of antioxidants prevent the increases in NA levels at the LC and changes in pain responses.

The clinical implications of the present study remain to be fully established, and in the future it will be important to evaluate pain modulation in patients with hydrocephalus. There are several constraints to available animal models of the disease. The profound structural alterations in the brain limit the studies of the manipulation of pain control centers, as their anatomic positions are severely altered. Behavioral pain tests are also hard to interpret due to the postural impairments of the animals; therefore, a correlation with biochemical markers of nociceptive neuronal activation, such as c-Fos, are necessary. The current advances in the field of functional neuroimaging in rodent brains may be useful to tackle the limitations of the current available methods.

## 4. Materials and Methods

All procedures were approved by the Institutional Animal Care and Use Committee of the Faculty of Medicine of the University of Porto (Porto, Portugal), and were performed in accordance with the European Community Council Directive (2010/63/EU) and the ethical guidelines for pain investigation [53]. Pathogen-free adult male Wistar rats (Charles River colony, France) were maintained under a controlled temperature (22 ± 2 °C) and light (12/12 h light/dark cycle, lights on between 8:00 h and 20:00 h) conditions with ad libitum access to food and water. The animals were allowed to acclimate to the housing facility for one week before the onset of the procedures. All experiments were conducted during the light phase. Subjective bias when allocating the animals to the experimental groups was minimized by arbitrarily housing the animals in pairs upon their arrival. Subsequently, the animals were randomly picked from the cage for each procedure. No a priori power analysis was performed. The sample sizes were based on common practice of the research group where, by default, six animals per group were used in experiments. In all the experiments, a blinded analysis was performed, as referenced below. There were no missing data or excluded animals, known as outliers. As for the anatomical studies, the atlas of Paxinos and Watson (2004) was used in the identification of rostrocaudal levels of the brains to ensure homogenous tissue sampling, and to determine the approximate distance to reference points such as the interaural line or the bregma of the sections analyzed, as described in our previous studies [18,54,55]. Briefly, sets of serially collected coronal sections of rat’s brain were previously counterstained with formol-thionin, and each section was identified regarding the distance to the anatomic references, such as the interaural line or the bregma [18,54,55].

### 4.1. Surgical Induction of Hydrocephalus

Wistar rats weighing 285–300 g (approximately 9 weeks old) were deeply anesthetized by an intraperitoneal (i.p) injection of a mixture of ketamine hydrochloride (0.06 g/Kg) and medetomidine (0.25 mg/Kg). The region of the head and neck was cleaned with Betadine^®^ solution and the atlanto-occipital membrane was exposed. Hydrocephalus was induced through percutaneous injection into the cisterna magna with a 27-gauge needle of a sterile kaolin suspension (0.05 mL of a 20% suspension). The injection was performed after the aspiration of cerebrospinal fluid (CSF) through the needle, confirming intracisternal access. The suspension was injected at a slow rate, and after completion of the injection the needle was left in place for a few seconds, to avoid reflow, before being slowly removed. Control rats underwent the same procedure but received sterile saline injection instead of kaolin. After stereotaxic injections, the animals were housed individually. All rats were closely observed during recovery and monitored daily to control general welfare.

### 4.2. Nociceptive Behavioral Evaluation (Formalin Test)

Four weeks after hydrocephalus induction, inflammatory nociception was evaluated by the formalin test as previously described [48]. Habituation was performed during the week that preceded the formalin test and the animals were handled by the researcher in the behavioral test room for 30 min every day [56]. During the formalin test, the animals were subcutaneously injected into the dorsal surface of the left hindpaw with 50 µL of a 5% formalin solution using a 27-gauge needle [48]. After the formalin injection, the animals were placed in individual Plexiglas chambers and a video camera located beneath the chamber floor was used to record the behavioral responses. The videos were posteriorly analyzed in 5 min epochs for 60 min by an observer that was blind as to the animal’s group. Two major behavioral categories were evaluated, as described previously [48]: (i) focused pain: time spent in focused pain-related activity—motor activity directed towards the injected paw, including biting, licking, and shaking of the injected paw; (ii) non-focused pain: time spent in non-focused pain-related activity—motor activity not directed towards the injected paw, but modified to protect the paw during movement [54]. The score for pain behavior was calculated as described previously [48].

Formalin injection produces a biphasic behavioral reaction with an initial phase during the first minutes post injection, followed by a quiescent period of around 10 min and a second phase lasting 20–40 min [55]. The first phase is related to the direct stimulation of nociceptors by the formalin activation of C fibers, whereas the second involves both inflammatory mechanisms and central sensitization within the dorsal horn. Between the two phases there is a quiescent period in which the animals present little behavior ascribed to nociception. Each of the phases was compared between the 2 experimental groups (control and hydrocephalic).

### 4.3. Vascular Perfusion and Material Processing for Immunohistochemical Analysis

Two hours after formalin injection, the animals were anaesthetized by i.p. injection with an overdose of sodium pentobarbital (65 mg/Kg of body weight). The animals were then placed in the supine position and the abdomen and thorax were opened to expose the heart. A catheter was then introduced into the ascending aorta and perfused with 200 mL of calcium-free Tyrode’s solution, followed by 1 L of fixative solution containing 4% paraformaldehyde in 0.1 M phosphate buffer (PB), pH 7.2. After perfusion, the brains and the lumbar spinal cord were removed, and immersed in fixative for a post-fixation period of 4 h followed by 30% sucrose in 0.1 M phosphate buffer, pH 7.2, overnight at 4 °C. The material was cut in a freezing microtome at 40 μm in coronal orientation. The brain and the spinal cord were serially cut and collected in 4 sets and stored in a cryoprotectant solution at −20 °C. One set of brain sections was used to determine the degree of periacqueductal dilatation, as described in detail below (Section 4.4 “Histological Analysis”). The others two sets of brainstem sections were used for the immunohistochemical reactions described below, namely tyrosine hydroxylase (TH), the enzyme catalyzing the rate-limiting step in catecholamine biosynthesis, and the oxidative stress marker (8-hydroxyguanosine; 8-OHdG). Regarding the spinal cord material, one set of spinal L4 sections was used to evaluate Fos expression and another for TH. Additional animals were used to study the expression of DBH at the spinal cord.

### 4.4. Histological Analysis

To evaluate the effectiveness of the induction of hydrocephalus, one section in every four was collected through the entire rostro-caudal extension of the Sylvius aqueduct and surrounding tissue at the PAG. The brain atlas of Paxinos and Watson [57] was used to allow the identification of the rostrocaudal level, namely using sets of coronal sections of brain of age-matched animals, counterstained with formol-thionin [18,54,55]. The sections were then serially mounted in gelatin-covered slides and stained by the formol-thionin method [58].

Photomicrographs of the sections were taken using a Zeiss^®^ light microscope using a high-resolution digital camera and measurement of the PAG and Aqueduct areas in mesencephalic sections was performed using ImageJ software (U.S. National Institutes of Health, Madison, WI, USA) (NIH, USA). The observer was not aware of the experimental group before accessing the photomicrographs of the sections.

### 4.5. Immunohistochemical Analysis of Fos Expression

The spinal cord was carefully washed with PBS 0.1 M and treated with 1% hydrogen peroxidase to inhibit the activity of endogenous peroxidase. The sections were then incubated with blocking solution (10% normal swine serum in 0.3% Triton-X 25% in phosphate buffer with 0.1 M glycine) before incubation with the primary antibody, a polyclonal anti-Fos antibody raised in rabbit (Calbiochem, San Diego, CA, USA, Cat. No. PC38), diluted at 1:20,000 in 0.1 M PBS containing 0.3% Triton X-100 (PBS-T) and 2% normal swine serum (NSS), for 48 h at 4 °C. After washing with PBS-T the sections were incubated for 1h with a swine biotinylated anti-rabbit serum (Dako, Copenhague, Danemark, EO353s) diluted in PBS-T containing 2% NSS. Sections were washed again and incubated for 1h in PBS-T containing the avidin-biotin complex (1:200; ABC; Vector Laboratories, Burlingame, CA, USA). After washing in 0.1 M Tris-HCl, pH 7.6, bound peroxidase was revealed using 0.0125% 3,3′-diaminobenzidine tetrahydrochloride (DAB; Sigma Aldrich, St. Louis, MO, USA) and 0.025% H_2_O_2_ in the same buffer. The sections were mounted on gelatine-coated slides, cleared in xylol and cover slipped with Eukitt (Sigma, St. Louis, MO, USA). Five spinal sections were randomly selected to count Fos-immunoreactive (IR) neurons in the dorsal horn (laminae I–V) using photomicrographs obtained with a Zeiss^®^ light microscope equipped with a high-resolution digital camera and the cell counter plugin from computer program Fiji. The observer was blind as to the animal’s experimental group.

### 4.6. Immunohistochemical Analysis of TH and DBH Expression

Brainstem sections were carefully washed with PBS 0.1 M and treated with 1% hydrogen peroxidase to inhibit the activity of the endogenous peroxidase. The sections were then incubated with blocking solution (10% NSS in 0.3% Triton-X 25% in phosphate buffer with 0.1 M glycine) before incubation with the primary antibody, a polyclonal anti-TH antibody raised in mouse (Sigma-Aldrich, St. Louis, MO, USA, Product No. T1299), diluted at 1:6000 in 0.1 M PBS containing 0.3% Triton X-100 (PBS-T) and 2% NSS, for 24 h at 4 °C. After washing with PBS-T, the sections were incubated for 1h with a rabbit biotinylated anti-mouse serum (Dako, Copenhague, Danemark, EO0354) diluted in PBS-T containing 2% NSS. Sections were washed again and incubated for 1 h in PBS-T containing the avidin-biotin complex (1:200; ABC; Vector Laboratories, Burlingame, CA, USA). After washing in 0.1 M Tris-HCl, pH 7.6, bound peroxidase was revealed using 0.0125% DAB (Sigma Aldrich, USA) and 0.025% H_2_O_2_ in the same buffer. The sections were mounted on gelatine-coated slides, cleared in xylol and cover slipped with Eukitt (Sigma Aldrich, St. Louis, MO, USA). For the immunohistochemical analysis of TH expression in the spinal L4 sections, the procedure was the same but the anti-TH antibody was diluted at 1:4000. We used all the sections encompassing the rostro-caudal extent of the LC in both experimental groups and sections of the L4 spinal segment.

For the DBH study, 5 sections of L4 segment from each animal were incubated with the primary antibody, a monoclonal anti-DBH antibody raised in mouse (Milipore, catalogue No. MAB308), diluted at 1:10,000 in 0.1 M PBS in 0.1 M PBS containing 0.3% Triton X-100 (PBS-T) and 2% of normal horse serum, for 24 h at room temperature. After washing with PBS-T, the sections were incubated for 1h with a horse biotinylated anti-mouse serum (Vector Laboratories, BA2000) diluted in PBS-T containing 2% normal horse serum. Sections were washed again and incubated for 1h in PBS-T containing the avidin-biotin complex (1:200; ABC; Vector Laboratories, Burlingame, CA, USA). After washing in 0.1 M Tris-HCl, pH 7.6, bound peroxidise was revealed using 0.0125% 3,3′-diaminobenzidine tetrahydrochloride (DAB: Sigma Aldrich, St. Louis, MO, USA) and 0.025% H_2_O_2_ in the same buffer. The sections were mounted on gelatine-coated slides, cleared in xylol and cover slipped with Eukitt (Sigma, St. Louis, MO, USA). The two hemiparts of the spinal dorsal horn were analyzed.

The methods used in this immunohistochemical analysis have previously been described for the brainstem [18,59] and spinal cord [24,60,61]. The observer was always blind as to the experimental group of each histology slide. Briefly, although the LC is easy to identify in sections immunostained for enzymes of the noradrenergic biosynthetic pathway, we used an additional set of sections that were previously prepared to guide the identification of noradrenergic cell groups in the rat’s brain in several studies of noradrenergic pain modulation of our research group, e.g., references [18,23,24,55]. These brain sections were immunoreacted for DBH and counterstained with formol-thionin, as described previously [18,24], allowing us to determine the rostrocaudal location of each brain section, based on the atlas by Paxinos and Watson [57]. Regarding the quantification of cathecolamine-synthetizing enzymes, photomicrographs of the material were taken using a Zeiss^®^ light microscope with a high-resolution digital camera. The photomicrographs included the left and right LC. Only the LC was included and not the surrounding areas, namely the SC area located ventrally, which collectively form the A_6_ noradrenergic cell group, the 2 hemiparts of the spinal dorsal horn (laminae I–V). Due to the high density of neurons and fibers in the LC [33,34], the counting of individual LC neurons cannot be performed accurately. In such situations, densitometric analysis is preferable [62]. It has been performed with TH immunoreaction of the LC in several studies [44,60,63,64]. In these cases, measurement of the optic density of the TH immunoreaction is used as an indication of the magnitude of TH expression in the LC [60]. A densitometric analysis was performed as described previously [39,61]. Briefly, sections were observed in a light microscope (Axioskop 40; Carl Zeiss, Jena, Germany), and the images were acquired using a high-resolution digital camera coupled to a computer. NIH Image J 1.52 software (National Institute of Health, Madison, WI, USA) was used for densitometric quantification. The mean percentage of pixels occupied by TH-immunostaining was calculated as described previously [24]. Normalization of the background preceded the computer generation of mean optical density. The method used to quantify the percentage of DBH positive pixels in the grey matter of the spinal L4 segment has been described in detail previously [55,57]. Briefly, the mean level of background was determined for each section using the region of interest analysis (ROI analysis) at the spinal dorsal horn. The mean of background level was determined for each section using ROI analysis of a small area without visible DBH fibers and a value of 5 standard deviations above the mean background level was used to set a threshold level for DBH-positive pixels.

### 4.7. Double Immunodetection of TH and 8-OHdG

To verify if noradrenergic neurons in the LC of hydrocephalic animals were more prone to oxidative stress, we performed a double immunohistochemical reaction for TH and 8-OHdG. Sections were treated with 1% borohydrate for 20 min, before incubation in a blocking solution containing 10% normal horse serum (NHS) in PBST with 0.1 M glycine for 2 h. Sections were then incubated with a primary polyclonal anti-8-OHdG raised in goat (ABCam, ab10802) diluted at 1:5000 in 0.1 PBS containing 0.3% Triton X-100 (PBS-T) and 2% NHS over 3 nights at 4 °C. After washing with PBS-T, the sections were incubated for 1 h with a horse biotinylated anti-goat serum (Vector Laboratories, BA9500) diluted in PBS-T containing 2% NHS. After being washed in a 2% NHS in PBST solution, the sections were incubated for 1 h at room temperature with Streptavidine Alexa 594 diluted at 1:1000 (Molecular Probes, Eugene, OR, USA). After washing with PBS-T and 2% NHS, the sections were incubated with a blocking solution containing 10% NHS in PBST with 0.1 M glycine, for 2 h. Sections were then incubated with a primary polyclonal anti-TH raised in mouse (Sigma-Aldrich, St. Louis, MO, USA, Product No. T1299), diluted at 1:6000 in 2% NHS in PBS-T, for 1 night at room temperature, followed by an Alexa 488 rabbit anti-mouse antibody diluted at 1:1000 (Molecular Probes) in a 2% NHS in PBS-T solution. The sections were mounted on gelatin-coated slides, coverslipped with buffered glycerol and analyzed in an Apotome Slider (Zeiss, Jena, Germany). Photomicrographs were taken using a high-resolution camera coupled to a computer. The numbers of neurons IR for 8-OhDG, TH or 8-OhDG + TH were bilaterally counted in sections encompassing the rostro-caudal extent of the LC. Only the LC was included and not the surrounding areas, namely the ventral SC. The percentages of TH-IR neurons that were also 8-OhDG were calculated in the 2 experimental groups to estimate the degree of oxidative stress in LC noradrenergic neurons. The observer was blind as to the animal’s experimental group.

### 4.8. Statistical Analysis

The statistical analysis of the results of the formalin test was performed using a two-way ANOVA followed by Sidak’s post hoc test using the computer program GraphPad Prism 6. The analysis of the results of the immunohistochemical studies were analyzed by an unpaired t-test for comparisons between the control and kaolin groups using the same computer program. As referred previously [48], the normality assumption was checked by inspection of the distribution of the variables both with q-q plots and histograms, but it must be acknowledged that the sample size limits the ability to detect departures from normality. *p*-values of <0.05 were considered statistically significant. The percentages of co-localization of 8-OhDG and TH-IR cells were calculated in both groups.

## Data Availability

All data presented in this study are available on request by contacting the corresponding author.
